# Is there an effect of intranasal insulin on development and behaviour in Phelan-McDermid syndrome? A randomized, double-blind, placebo-controlled trial

**DOI:** 10.1038/ejhg.2016.109

**Published:** 2016-08-31

**Authors:** Renée J Zwanenburg, Gianni Bocca, Selma A J Ruiter, Jan H Dillingh, Boudien C T Flapper, Edwin R van den Heuvel, Conny M A van Ravenswaaij-Arts

**Affiliations:** 1 University of Groningen, University Medical Centre Groningen, Department of Genetics, Groningen, The Netherlands; 2 University of Groningen, University Medical Centre Groningen, Beatrix Children's Hospital, Department of Paediatrics, Groningen, The Netherlands; 3 De Kinderacademie Groningen, Centre of Expertise for Child Development Care and Research, Groningen, The Netherlands; 4 University of Groningen, University Medical Centre Groningen, Department of Clinical Pharmacy and Pharmacology, Groningen, The Netherlands; 5 Department of Mathematics and Computer Science, Eindhoven University of Technology, Eindhoven, The Netherlands

## Abstract

Phelan-McDermid syndrome (PMS) or 22q13.3 deletion syndrome is a rare neurodevelopmental disorder with at least 60 children and 35 adults diagnosed in the Netherlands. Clinical features are moderate to severe intellectual disability and behavioural problems in the autism spectrum. Other researchers had observed a beneficial effect of intranasal insulin on development and behaviour in a pilot study in six children with PMS. To validate this effect, we conducted a randomized, double-blind, placebo-controlled clinical trial using a stepped-wedge design. From March 2013 to June 2015, 25 children aged 1–16 years with a molecularly confirmed 22q13.3 deletion including the *SHANK3* gene participated in the clinical trial for a period of 18 months. Starting 6 months before the trial, children were systematically assessed for cognitive, language and motor development and for adaptive, social and emotional behaviour every 6 months. The second, third and fourth assessments were followed by daily nose sprays containing either intranasal insulin or intranasal placebo for a 6-month period. A fifth assessment was done directly after the end of the trial. Intranasal insulin did not cause serious adverse events. It increased the level of developmental functioning by 0.4–1.4 months per 6-month period, but the effect was not statistically significant in this small group. We found a stronger effect of intranasal insulin, being significant for cognition and social skills, for children older than 3 years, who usually show a decrease of developmental growth. However, clinical trials in larger study populations are required to prove the therapeutic effect of intranasal insulin in PMS.

## Introduction

Phelan-McDermid syndrome (PMS, OMIM 606232) is caused by a deletion on chromosome 22q13.3 that results in a neurodevelopmental disorder. There are at least 60 children and 35 adults diagnosed with this syndrome in the Netherlands. The main clinical features of PMS are intellectual disability and behavioural problems in the autism spectrum.^1^ The behavioural profile is characterised by a general delay in adaptive behaviour, which is seen as deficits in social communication.^2^ In a recent study, we showed less favourable developmental growth in PMS patients after the age of 3 years, with a slow but steady increase in developmental abilities up to that age that is followed by decreased or stagnated developmental growth after.^3^

The neurodevelopmental features in PMS are mainly caused by a deletion of the *SHANK3* gene (OMIM 606230).^4^ Mutations in this gene have been reported in humans with intellectual disability and with autism (reviewed by Leblond *et al.*),^5^ and have been shown to cause deficits in social interaction and learning in animal models.^6, 7^ SHANK3 is a scaffold protein located at axon terminals and postsynaptic densities of neurons that has an important role in synaptic maturation and neuronal signalling.^8, 9^ Neurons derived from induced pluripotent stem cells of PMS patients showed reduced SHANK3 mRNA and protein expression, a decreased number of excitatory neurotransmitter-receptors and fewer excitatory synapses.^9^ As the molecular mechanisms underlying the syndrome are uncovered, research is gradually focusing on developing therapeutic strategies at the biological level. One strategy is the administration of insulin-like growth factor 1 (IGF-1), which has been reported to reverse deficits in synaptic signalling in heterozygous *Shank3*-deficient mice.^10^ In a randomized placebo-controlled crossover pilot study, nine children with PMS were treated with daily intraperitoneal IGF-1 injections over a period of 12 weeks. The results showed an improvement of social impairment and restrictive behaviour.^11^ A non-invasive therapeutic strategy is treatment with intranasally administered insulin. Intranasal application leads to uptake of insulin in the cerebral spinal fluid (CSF), bypassing the blood–brain barrier.^12^ Insulin administered by this route has been shown to improve memory, general cognition, functional abilities and mood in healthy adults, adults with Alzheimer's disease and adults with minimal cognitive impairment without causing systemic effects on blood glucose levels or other serious adverse events (SAEs; reviewed by Shemesh *et al.*).^13, 14^ Schmidt *et al.* hypothesised that intranasal insulin could compensate for the cognitive deficits in PMS and conducted an exploratory non-placebo-controlled study in six children with PMS.^15^ They reported an improvement of motor activity, cognitive function, nonverbal communication and autonomy after treatment with intranasal insulin, without an effect on blood glucose levels or SAEs. However, replication of these effects in a clinical trial with a larger number of participants is needed.

The aim of our study was to evaluate the effect of intranasal insulin on development and behaviour in a larger group of children with PMS using a randomized, double-blind, placebo-controlled clinical trial.

## Subjects and methods

### Study design and subjects

**Figure 1:**
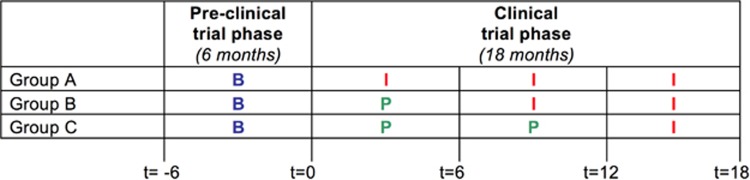
Study design. Trial phases, study groups and study periods. B, baseline; I, intranasal insulin; *P,* intranasal placebo. *t*=time point (in months) at which development and behaviour were assessed.

The study is a monocenter, randomized, double-blind, placebo-controlled trial with a stepped wedge design including three study groups.^16^ Before the clinical trial, we statistically evaluated several different trial designs (parallel group design, matched-pairs design, wait-list design and stepped wedge design) and found the stepped wedge design showed the highest power for testing treatment effect.^16^ The stepped wedge design is especially appropriate for studies in which the intervention is expected to do more good than harm, a situation in which it is considered unethical to withhold treatment from a subgroup of participants (as in parallel group design) or to only provide treatment to all participants if the effect has been proven to be effective (as in wait-list design). With the stepped wedge design, the change from intranasal placebo to intranasal insulin occurs at different time points for each study group (Figure 1).

The study was performed between March 2013 and June 2015. Patients were recruited from a group of 38 Dutch children with PMS who had been diagnosed in the Clinical Genetics department of the University Medical Centre Groningen or referred to our centre from other hospitals in the Netherlands. Inclusion criteria were a molecularly confirmed 22q13.3 deletion including *SHANK3*, a calendar age between 12 months and 18 years, and having parents who understand and speak Dutch (necessary because of the nature of the assessments). Children with severe (perinatal) brain damage or with a metabolic or neuromuscular disease were excluded because development and behaviour could be significantly influenced by these factors and less dependent on their SHANK3 deficiency, the hypothetical target of the treatment. Of the 38 children, two were lost to follow-up before the start of the trial, parents of 10 children decided not to participate in the trial and we excluded one child with severe brain damage. Ultimately, 25 children were included in the trial.

Basic characteristics of participants, treatment exposure and safety endpoints were collected for all participants. The primary objective was to investigate whether intranasal insulin leads to a greater increase in the level of general developmental functioning compared with the change in developmental functioning without insulin. The secondary objective was to investigate whether intranasal insulin leads to an improved functioning of adaptive and social-emotional behaviour.

### Treatment allocation

The study period consisted of a pre-clinical trial phase and a clinical trial phase (Figure 1). During the pre-clinical trial phase, patients and their parents visited our expert clinic for Rare Chromosome Disorders. A patient history was taken and a physical examination was performed by a paediatrician (GB) to evaluate potential health issues. Patients were randomly allocated to one of the study groups. Randomisation was done by the Department of Clinical Pharmacy and Pharmacology of the University Medical Centre Groningen using a restricted randomisation method with permuted blocks of three groups (A, B and C). During the clinical trial phase, following the pre-treatment assessment (*t*=0 months), participants started with either intranasal insulin (group A) or intranasal placebo (group B and C) according to schedule (Figure 1). Study investigators, parents and participants were blinded for treatment allocation.

### Study medication and placebo

According to schedule, patients received a set of nose sprays with either insulin or placebo solution each 6-month period. The recombinant human insulin solution for this study consisted of the licensed parenteral drug Humuline Regular 100 IU/ml (Eli Lilly Nederland B.V., Houten, The Netherlands). The dosage was based on the pilot study of Schmidt *et al.* 2009.^15^ Our Department of Clinical Pharmacy and Pharmacology repackaged this product in multi-dose nasal spray bottles (Aeropump, Hochheim am Main, Germany). One spray was 0.1 ml, containing 10 IU of insulin. The composition and packaging of the placebo solution was identical to the insulin buffer solution. In the placebo solution, insulin was replaced by human albumin (Albuman 200 g/l, Sanquin, Amsterdam, The Netherlands) to a concentration of 3.47 mg/ml.

In previous studies, the maximum dose that had been safely administered to adults was 160 IU/day (~2.5 IU/kg/day)^17^ and to children 20–60 IU/day (0.5–1.5 IU/kg/day).^15, 18^ The daily dose for our study was calculated so that the maximal safe dose from previous studies was maintained, but maximal levels in the CSF would be achieved. Because head circumference in children corresponds to their brain volume,^19^ we calculated the insulin dose based on both estimated body weight^20^ and head circumference^21^ (a proxy for cranial volume) to find the optimal therapeutic dose (Table 1). The medication was administered twice a day: just before breakfast and dinner. When two sprays were scheduled, parents administered one spray to each nostril. The medication was accompanied by administration instructions for the parents.

### Safety

Several independent studies have shown that intranasal insulin administration did not alter systemic insulin concentration or glucose levels in children and adults.^13, 15, 18^ Therefore, peripheral glucose or insulin levels were not measured in study participants. Safety and tolerability were evaluated based on both expected and unexpected adverse events and SAEs as reported by parents to the investigators by email, phone and through their participant diaries. Previously reported, and therefore expected, adverse events were transitory nasal irritation, rhinitis, spontaneous nose bleeds and headache.^13^ As the placebo buffer solution consisted of the same components as the insulin buffer solution, the potential adverse effects of the placebo solution were expected to be similar.

### Outcome measures

To measure the effect of intranasal insulin, development and behavioural functioning were assessed at 6-month time intervals by different tests (see next section). The rates of improvement of development and behaviour in the treatment period were then compared to the rates of improvement in the pre-treatment period.

The results from the *t*=−6 and *t*=0 month assessments of the pre-clinical trial phase were used to determine baseline changes in general development and behaviour.

In the clinical trial phase, each subsequent treatment period consisted of 6 months at the end of which time development and behaviour were assessed (*t*=6, *t*=12 and *t*=18 months). Immediately after each assessment, participants switched to a new set of nose sprays (Figure 1). Once started on intranasal insulin, participants remained on insulin until the end of the trial.

### Assessment of development and behaviour

Two licensed clinical child psychologists with professional experience, intensively trained in the administration of the instruments used in this study and experienced in working with children with special needs, assessed children at home or in their school environment. General development, our primary outcome parameter, was assessed by the Bayley Scales of Infant and Toddler Development,^22^ third edition, adapted and validated for the Dutch population (Bayley-III-NL),^23^ or Wechsler Preschool and Primary Scale of Intelligence,^24^ third edition, Dutch version (WPPSI-III-NL)^25^ depending on the developmental age of the children. Bayley-III-NL and WPPSI-III-NL outcomes are expressed in raw scores that were converted to a developmental age equivalent (DAE) as previously described.^3^

Behaviour, our secondary outcome parameter, was assessed by two questionnaires applied in interview form: the Vineland Screener 0–6 and the *Experimentele Schaal voor de beoordeling van het Sociaal-Emotionele Ontwikkelings Niveau* (ESSEON). The Vineland screener 0–6, which is based on the American short version of the Vineland Adaptive Behavior Scales (VABS),^26^ was used to cover four domains of adaptive behaviour portrayed in the original Expanded Version of the VABS: communication, social, daily and motor skills. The Vineland Screener has been validated for Dutch children aged 0–6 years and for children between 1 and 18 years with a developmental delay.^27^ Raw scores of each subscale were converted to a DAE. The ESSEON is a Dutch instrument that determines the level of social and emotional development of children from 0 to 14 years of age.^28^ It is a behaviour rating scale based on interviewing adults with knowledge of the child's behaviour, resulting in a DAE for each subscale. There is some overlap between the Vineland Screener and the ESSEON with respect to aspects of adaptive behaviour-like social and communicative abilities, but these questionnaires are complementary with respect to emotional development.

In addition to our primary and secondary objectives, we evaluated behavioural problems, which were assessed using two questionnaires: the BRIEF-P^29^ and the Child Behavior Checklist (CBCL/1.5-5).^30^ Methods and results are described in Supplementary File.

### Statistics

The statistical analysis for treatment effect was conducted using a subject-specific random coefficients (linear mixed) model which has been described previously (SWD-S3).^16^ It is based on comparing the change in DAE (with respect to change in biological age) for each individual subject from the control period, for example, the period of baseline and/or placebo, with the period in which children received intranasal insulin. A *P*-value<0.05 was considered to be statistically significant. The intention-to-treat analysis was performed with all participants and in all periods. The on-treatment analysis was performed based on all periods in which participants had a >90% treatment adherence, which was evaluated by returned medication and participant diaries. A subgroup analysis was performed for children >36 months who usually show a decrease or stagnation of developmental growth^3^ to see whether this has implications for the treatment effect.

### Ethical considerations and trial registration

The Institutional Medical Ethical Review Board of the University Medical Centre Groningen approved this study (protocol ID 2012/329) and all parents provided written informed consent. The study was conducted according to the principles of the Declaration of Helsinki (version 59th WMA General Assembly, Seoul, October 2008) and in accordance with the Medical Research Involving Human Subjects law (WMO, 26 February 1998, version 1 March 2006). The clinical trial (local protocol code number 2013.2015) was registered at the national competent authority (NL-41213-042-12), the Netherlands Trial Register (NTR 3758) and the EU Clinical Trial Register (2012-002873-77).

## Results

### Subjects

Twenty-five children participated in the trial, 6 males and 19 females, of whom 23 fully completed the treatment period (Table 2 and next section). Clinical characteristics of these patients have been described previously (patients 1, 2, 4, 5, 8, 9, 11–14, 16, 17, 19, 20, 22, 23 and 26–34).^3^ Nine children were randomised in group A, 8 in group B and 8 in group C.

### Treatment adherence and Safety

In general, treatment adherence was high in this study. One child did not receive sufficient medication during periods 2 and 3 due to social circumstances (inability to properly organise the spray application), one child received ~2/3rds of the daily dose in period 3 on the parents' own initiative and one child received ~80% of the medication in period 3 because the parents forgot to administer the medication several times.

Two children did not finish the whole-treatment period of the study due to non-SAEs. One child suffered from abdominal pain at the end of period 2 (under placebo) and subsequent paediatric evaluation suggested mild pancreatitis with elevated amylase levels. A relation with the nasal spray was not likely as this child had been using this batch of spray for at least 5 months and none of the other children had similar complaints. Nonetheless, the parents decided to discontinue with the study. Another child used the nasal sprays until the fourth week of period 3, but had suffered from recurrent nosebleeds beginning in period 2 so that the parents decided to discontinue the medication for a while but then forgot to restart treatment.

No SAEs or suspected serious adverse reactions have been reported. Expected adverse events such as nosebleeds and irritation of the nasal area were frequently reported (12 of 25 children). These were limited to 1 or 2 days and usually occurred only once per period in both placebo and insulin groups. Most other adverse events reported, like upper airway infections and gastro-enteritis, are common in children and occurred in both placebo and insulin groups.

### Primary and secondary endpoints

Table 3 shows the results of the intention-to-treat analyses on general and behavioural development, displaying the mean changes in DAE for the pre-treatment period (control rate), the additional increases in DAE in the treatment period (insulin effect) and the changes in DAE combined (total rate).

In the pre-treatment period, the mean change in DAE ranged from 0.4 to 1.8 months per 6 months for the five domains of general development (Bayley-III-NL). Intranasal insulin resulted in an additional increase of the DAE for cognition (0.6 months per 6 months), receptive language (0.6 months per 6 months) and fine motor skills (0.4 months per 6 months). Although this additional effect of intranasal insulin for these domains was not significant itself, the total change in DAE with intranasal insulin was statistically significant, as illustrated by the 95% confidence intervals (Table 3). The mean contribution of intranasal insulin to the total change in DAE was 59, 38 and 47% for these respective domains. The change in DAE of expressive language and fine motor skills of the pre-treatment period was already significant and the additional effect of intranasal insulin was approximately zero.

There was also a positive effect of intranasal insulin observed on all four domains of adaptive behaviour (Vineland Screener 0-6) and on social development (ESSEON). In the pre-treatment period, the change in DAE ranged from 0.1 to 2.4 months per 6 months, except for social skills of adaptive behaviour. Intranasal insulin resulted in an additional increase of the DAE for all domains except emotional development, including social skills of adaptive behaviour (Table 3). This additional increase in DAE ranged from 0.6 to 1.4 months per 6 months. Although this additional effect of intranasal insulin was also not statistically significant itself, the total change in DAE with intranasal insulin was significant in contrast to the change in DAE without insulin treatment. The mean contribution of intranasal insulin to the total change in DAE was >80% for adaptive behaviour and 19% for social development. The on-treatment analysis did not result in different outcomes for development or behaviour (data not shown).

The subgroup analysis of children aged >36 months is shown on the right in Table 3. Compared with the whole-study group, a lower change in DAE in the pre-treatment period was shown for 8 of 11 domains, and a higher additional increase in DAE with insulin for 6 of 11 domains (difference of 0.1–0.4 months per 6 months). Moreover, a statistically significant effect of insulin was seen in this subgroup for the domains cognition (increase in DAE 0.8 months per 6 months, *P=*0.046) and social skills (1.5 months per 6 months, *P=*0.042). The contribution of intranasal insulin to the total change in DAE was 80% for cognition and >100% for social skills because the control rate was negative. Remarkably, the observed additional effect of intranasal insulin on DAE in this subgroup was positive for the domain of expressive language (increase in DAE 0.3 months per 6 months, 21% contribution to the total change of DAE), an effect that was not seen in the total study group (0.0 months per 6 months).

Supplementary material 1 shows the results of the intention-to-treat analyses for behavioural problems for the whole group as assessed by the BRIEF-P and the CBCL/1.5-5. None of these effects were statistically significant or clinically observable.

## Discussion

The aim of this study was to evaluate whether the previously reported positive effect of intranasal insulin on development and behaviour in children with PMS could be confirmed in a randomized, double-blind and placebo-controlled clinical trial. Our study shows a positive observed effect of intranasal insulin on psychomotor and behavioural development. However, this effect of intranasal insulin was not strong enough to reach statistical significance in our small group of patients, but some statistically significant positive effects were seen in the subgroup of children older than 3 years of age.

Obtaining significant results from clinical trials in a small cohort of patients is a challenge, especially in a clinically variable disorder like PMS, and there are a number of factors that need to be considered that may have impacted the size of the signals we measured.

First of all, our initial power analysis assumed that children with PMS would develop at a rate of 28% of typical development (1.7 months per 6 months) based on data from four children with PMS.^16^ However, as we recently showed in a study of 34 children, development in PMS is not a linear process and development stagnates after the age of 3 years.^3^ For most of the developmental domains that were analysed in the present study, the developmental rate under normal circumstances was therefore slower than expected, making an additional 15–45% treatment effect of intranasal insulin based on the previously expected higher basal rate too optimistic, further meaning that smaller effects of intranasal insulin on DAE are hard to detect in this sample size. Nonetheless, we saw an effect of insulin: while the DAE without insulin did not significantly improve over time for most domains, the increase of DAE with intranasal insulin treatment (total rate, Table 3) did reach statistical significance on all domains except emotional development. Moreover, this additional effect of intranasal insulin contributed 19 to >80% of the total increase in DAE. This might suggest that intranasal insulin itself has a limited and non-significant effect on development, but when the effect of insulin is added to the baseline development, it may contribute to a positive increase in development.

Second, the children who participated in the clinical trial vary widely in calendar age, deletion size and in their level of developmental functioning. Theoretically, this could result in a different therapeutic effect for subgroups. We previously found that children with PMS younger than 3 years of age have a higher natural developmental rate than children older than 3 years of age, who increasingly deviate from the typical developmental rate.^3^ Interestingly, subgroup analyses in children older than 36 months suggested a stronger effect of intranasal insulin in this group and showed significant results for the effect of intranasal insulin for the domains of cognition and social skills (Table 3). Thus, younger children might benefit less from intranasal insulin to reach their maximal capacity for development, whereas older children who do not develop at their maximal capacity could benefit more. Further studies are needed to support this observation. Unfortunately, the study group was too small for a subgroup analysis on the effect of insulin in children with small (<250 kb) versus larger deletions and more research is required to identify those children who might benefit most from intranasal insulin.

Third, at least 7 out of the 25 children received a dose that was too low compared with that received by other children with the same head circumference. We had to compromise between the maximal safe dose from previous studies (based on total body weight) and the maximal levels we could obtain in the CSF (based on a proxy for brain volume) because a safety study of intranasal insulin based on brain volume was not available. If we had determined the daily dose based on the maximal safe CSF levels, seven children would have received a higher dose, especially the children in the youngest age group (10–15 kg). Another challenge with intranasal application of insulin is that we cannot determine the exact amount of fluid administered in the nasal cavity nor the actual uptake in the CSF or the distribution and localisation in the brain tissue itself.

Finally, even though the treatment adherence appeared to be very high, we relied on patient diaries and returned medication, which are less objective. Still, we think that our estimations of treatment adherence were reasonable since administration of the nose-spray had a relatively low burden, limited adverse reactions and parents were highly motivated to participate in the study.

Despite our modest results, we do think that intranasal insulin could be a therapeutic strategy in children with PMS, based on the function of insulin in the brain. Insulin and IGF-1 function as neurotransmitters in the brain and both bind cerebral insulin, IGF-1 and insulin/IGF-1 hybrid receptors with different affinity (reviewed by Fernandez and Torres-Alemán.).^31^ The insulin/IGF-1 signalling pathway is involved in dendritic spine and synapse formation upon activation of the PI3K-Akt-mTOR signalling pathway.^32^ In PMS, dendritic spine formation and synaptic signalling are impaired because of SHANK3 haploinsufficiency as shown in Shank3-deficient animal models.^6, 33^ Excitingly, IGF-1 was shown to rescue synaptic transmission impairments of neuronal stem-cells of PMS patients by decreasing the number of synapses with SHANK3 and increasing the number of synapses with PSD-95.^9^ In addition, insulin has been shown to stimulate expression of the postsynaptic scaffolding protein PSD-95 in rat neurons.^34^ These studies therefore suggest that the effects of SHANK3 haploinsufficiency can be compensated by insulin/IGF-1 signalling via up-regulation of PSD-95 and increasing the number of PSD-95 containing synapses. For future studies, it would also be interesting to investigate the (short-term) effect of intranasal insulin in the brain by measuring event-related potentials or using functional magnetic resonance imaging. Although these techniques have not yet been standardized or validated for testing the effect of medication on development and behaviour in children with developmental delay, they would be very interesting to use in follow-up experiments.

A pilot study with daily intraperitoneal IGF-1 injections in patients with PMS showed a beneficial effect on social impairment and restrictive behaviour.^11^ A phase-2 study is currently ongoing in the USA to validate the effect of IGF-1 in patients with PMS (www.ClinicalTrials.gov ID: NCT01970345). Interestingly, intranasally applied IGF-1 can also be transported to the CSF/brain,^35^ but no clinical trials have been conducted with intranasal IGF-1 in patients with PMS.

In conclusion, the application of intranasal insulin is a non-invasive, safe, easy and interesting therapeutic approach to improve intellectual and behavioural development in children with PMS. Our preliminary results show that it increased the level of developmental functioning, especially in children older than 3 years, who usually show a stagnation of development. Most results were not statistically significant in our small study group but may be considered clinically relevant. Clinical trials in larger study populations are required to prove the true effect of intranasal insulin on development and behaviour, and might be able to determine which individuals would benefit most from it. In addition, future studies should focus on improving the local side-effects by adapting the insulin formulation and by studying the effects of intranasal insulin in the brain by using Shank3-deficient animals, a model for PMS. Still, we think that treatment with medication in children with complex disorders like PMS should only be part of the total and individualised treatment plan. Therapeutic support (speech, occupational, physical and behavioural therapy) with attention to individual needs are as important, if not more important, to optimise health and wellbeing in individuals with PMS.

## Figures and Tables

**Table 1:** Biometric characteristics and dose of study medication

	*Body*	*Cranial*		*Dose based on*		
*Age*	*weight*^a^	*volume*^b^	*Dose*	*Weight*	*Volume*	*Administration*
*(years)*	*(kg)*	*litres (l)*	*IU/day*	*IU/kg/day*	*IU/l/day*	*no of sprays*^c^
1–3	10–15	1.0–1.1	20	1.3–2.0	18.2–20.0	2 dd 1
3–9	15–30	1.1–1.25	30	1.0–2.0	24.0–27.3	1 dd 2 + 1 dd 1
9–18	30–65	1.25–1.5	40	0.6–1.3	26.7–32.0	2 dd 2

a Estimated body weight (based on ref. 18).

b Estimated cranial volume (ref. 19).

c One nasal spray is 0.1 ml and contains 10 IU.

**Table 2:** Baseline characteristics of the study group at *t*=0

n=*25*	*Mean (SD)*	*Median*	*Range*
Age (months)	82.5 (47.8)	75.0	13–189
Deletion size (Mb)	3.9 (2.5)	3.4	0.18–7.8
Cognitive DAE at start (months)	18.0 (11.1)	14.0	5.1–53
Mean weight at start (kg)	25.3 (13.8)	24.0	8–60
Head circumference at start (cm)	50.8 (3.2)	50.5	44.7–56

**Table 3:** Results of the intention-to-treat analysis for general and behavioural development

	*Control rate*		*Insulin effect*		*Total rate*		*Control rate*		*Insulin effect*	
	*All ages*		*All ages*		*All ages*		*Age >36 months*		*Age >36 months*	
	*C*	P-value	*C*	P-value	*C*	*95% CI*	*C*	P-value	*C*	P-value
B: cognition	0.40	0.30	0.57	0.11	0.97	(0.36; 1.58)	0.19	0.64	0.75	**0.046**
B: receptive language	1.02	0.02	0.63	0.13	1.64	(1.04; 2.25)	0.87	0.05	0.60	0.14
B: expressive language	1.35	0.002	0.05	0.92	1.39	(0.85; 1.94)	1.09	0.02	0.29	0.54
B: fine motor function	0.47	0.18	0.41	0.26	0.87	(0.38; 1.37)	0.30	0.44	0.56	0.15
B: gross motor function^a^	1.82	0.01	−0.07	0.92	1.75	(1.12; 2.38)	1.82	0.004	−0.01	0.99
V: communication skills^b^	0.11	0.80	0.57	0.19	0.68	(0.004; 1.35)	0.14	0.76	0.56	0.32
V: social skills	−0.12	0.88	1.41	0.06	1.29	(0.11; 2.47)	−0.45	0.57	1.54	**0.042**
V: daily skills	0.14	0.78	0.84	0.14	0.97	(0.33; 1.61)	0.04	0.94	1.10	0.07
V: motor skills	0.30	0.64	1.21	0.09	1.51	(0.62; 2.40)	0.07	0.91	1.28	0.10
E: social development^a^	2.39	0.13	0.56	0.76	2.96	(1.08; 4.83)	2.31	0.18	0.93	0.64
E: emotional development^a^	0.50	0.61	0.07	0.95	0.57	(−0.59; 1.74)	0.47	0.66	0.05	0.97

Abbreviations: B, Bayley-III-NL; V, Vineland Screener 0-6 and E=ESSEON.

a The random coefficients model was reduced to a random intercept model since the variability in the slope was estimated to be zero for ‘all ages' and ‘age >36 months' and

b for ‘age >36 months' only. All ages *n*=25, Age >36 months *n*=21. The coefficient (C) is an estimate of the change in DAE in months per 6 months for the pre-treatment period (control rate) and treatment period (insulin effect). Observed insulin effects that show a clinical increase in DAE are underlined. The *P*-value (*P*) is calculated for the difference between the DAE at the beginning and the end of these respective periods. *P*<0.05 is considered statistically significant. Significant *P*-values of insulin effects are bold. The confidence interval (CI) represents the distribution of the individual coefficients.
