# Supplementary material for: Is there an effect of intranasal insulin on development and behaviour in Phelan-McDermid syndrome? A randomized, double-blind, placebo-controlled trial
Source: Eur J Hum Genet. 2016 Aug 31;24(12):1696–701. doi: 10.1038/ejhg.2016.109 (PMC5117914; doi:10.1038/ejhg.2016.109)
Supplement: Supplementary Information [file ejhg2016109x1.pdf]

Supplement 1. Results of the intention-to-treat analysis for behavioural problems

Methods

In addition to our primary and secondary objectives, we evaluated behavioural problems, which were assessed using two questionnaires: The Behavior Rating Inventory of Executive Function–Preschool Version (BRIEF-P) and the Child Behavior Checklist (CBCL/1.5-5). The BRIEF-P rates executive function problems within the context of the everyday environments[1]. The CBCL/1.5-5 covers an empirical range of behavioural, emotional and social function problems. We used the Dutch version of the CBCL/1.5-5 with international, multicultural norms[2]. Raw scores of both the BRIEF-P and CBCL/1.5-5 were converted in t-scores. A BRIEF-P t-score <60 is considered within the normal range, between 60 and 65 borderline clinical and >65 as clinical range. A CBCL/1.5-5 t-score <64 is considered within the normal range, between 65 en 69 borderline clinical and >70 as clinically relevant. A decrease in t-score represents a reduction of behavioural problems.

The effect of treatment was analysed by calculating the difference in Developmental Age Equivalents (DAEs) or t-scores (Brief-P and CBCL/1.5-5) between two consecutive assessments, each 6 months apart.

Results

A limited reduction in t-scores was seen for the domains of inhibition, emotion regulation and attention and these reductions were neither clinically nor statistically significant (Table S1).

Table S1: Results of the intention-to-treat analysis for behavioural problems

	**Control Rate**		**Insulin Effect**		**Total Rate**	
	C	***p***	***C***	***p***	***C***	***95% CI***
B-P: Inhibition	0.53	0.59	-1.97	0.07	-1.44	[-2.65; -0.24]
B-P: Flexibility	-2.36	0.06	1.47	0.23	-1.20	[-2.36; 0.57]
B-P: Emotion regulation	-0.37	0.77	-0.19	0.87	-0.56	[-2.24; 1.12]
B-F: Memory	-1.89	0.06	0.63	0.52	-1.26	[-2.47; -0.05]
B-P: Planning	-3.26	0.03	1.30	0.39	-1.96	[-3.83; -0.09]
C: Anxiety[1]	-0.80	0.09	0.36	0.44	-0.44	[-1.03; 0.15]
C: Withdrawn	-3.15	<0.001	1.34	<0.001	-1.82	[-2.95; -0.68]
C: Attention	0.04	0.97	-1.17	0.21	-1.13	[-2.12; -0.14]
C: Aggression	-1.18	0.07	0.23	0.70	-0.95	[-1.83; 0.08]

The coefficient (C) provides an estimate of the change in t-score in points per 6 months for the control period (Control Rate) and insulin period (Insulin effect). The p-value (p) is calculated for the difference between the DAE at the beginning and the end of these respective periods. $p<0.05$ is considered statistically significant. The confidence interval (CI) represents the distribution of the individual coefficients. B-P=BRIEF-P and C=CBCL1.5-5.

[1]The random coefficients model was reduced to a random intercept model since the variability in the slope was estimated to be zero.

References

1 Gioia GA, Espy KA, Isquith PK. *Behavior Rating Inventory of Executive Function*. Psychological Assessment Resources: Odessa, FL, 2002.

2 ASEBA. Achenbach System of Eperically Based Assessment. www.aseba.nl.
